# First person – Maize Cao

**DOI:** 10.1242/dmm.049827

**Published:** 2022-09-13

## Abstract

First Person is a series of interviews with the first authors of a selection of papers published in Disease Models & Mechanisms, helping researchers promote themselves alongside their papers. Maize Cao is first author on ‘ Transcriptional targets of amyotrophic lateral sclerosis/frontotemporal dementia protein TDP-43 – meta-analysis and interactive graphical database’, published in DMM. Maize is a PhD student in the lab of Dr Emma Scotter at University of Auckland, New Zealand, Auckland, investigating downstream events of RNA-binding proteins involved in neurodegenerative diseases.


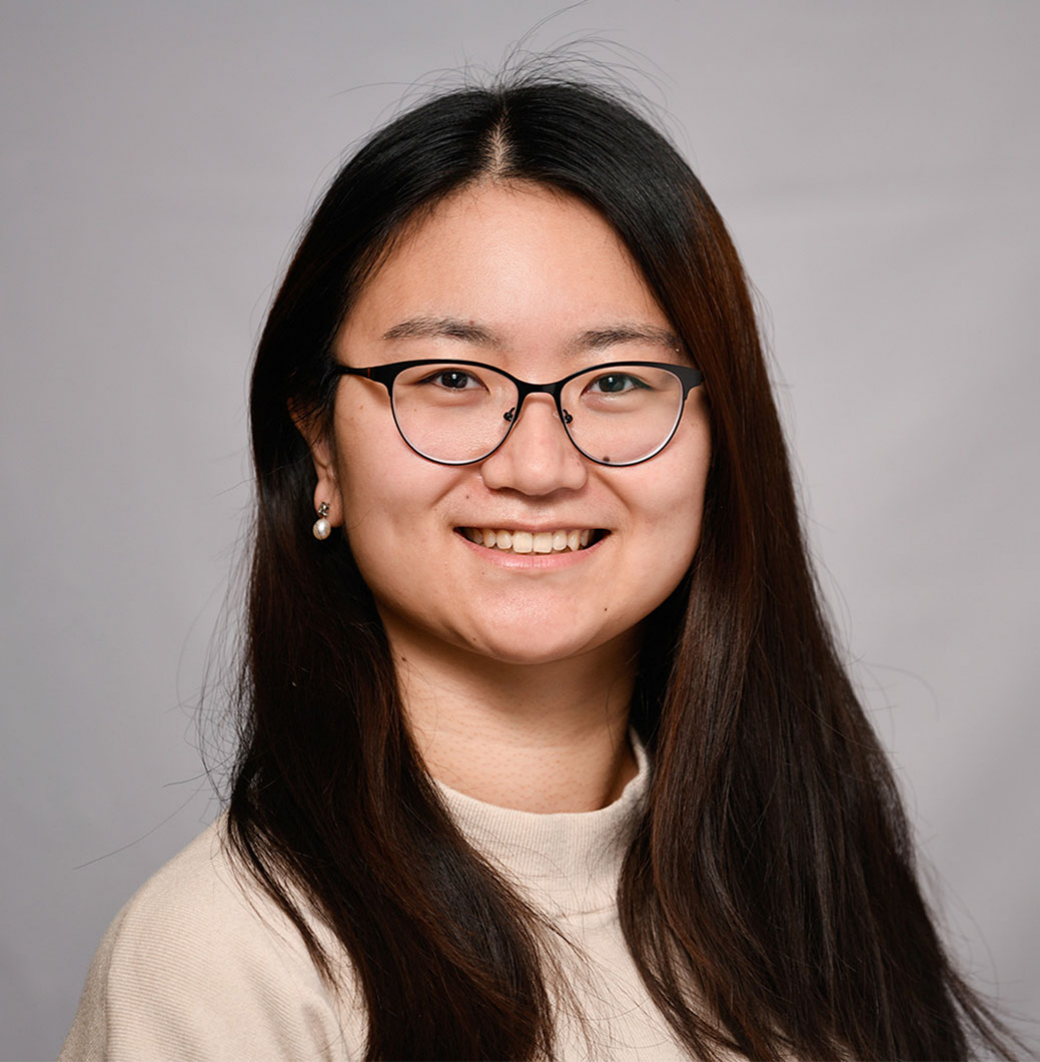


**Maize Cao**

**How would you explain the main findings of your paper to non-scientific family and friends?**

Amyotrophic lateral sclerosis (ALS) and frontotemporal dementia (FTD) are debilitating neurodegenerative diseases. The causation of disease is difficult to untangle, but a common denominator is the dysfunction of a protein called TDP-43 (encoded by *TARDBP*). TDP-43 binds to DNA and RNA, which are critical to gene expression. Every cell type has a special function that is determined by RNA transcripts. This is what makes cells unique from one another. When TDP-43 function is impeded, the interaction with RNA transcripts is lost, affecting the gene expression of the cell. This can lead to a change in biological function, which can be detrimental. By combining published studies of TDP-43 loss of function that have used RNA sequencing to analyse gene expression, we can determine what RNA transcripts (or parts of transcripts) have been changed in various cell types. Identifying these changes are essential for unravelling the functions of TDP-43 and shed light on the cause of disease.

**What are the potential implications of these results for your field of research?**

The impairment of gene expression and splicing regulation are now widely accepted features of ALS with TDP-43 proteinopathy, but how the specific complement of *TARDBP* mRNA targets drives pathogenesis and disease phenotype remains unclear. By assessing *TARDBP*/*Tardbp* mRNA targets in human and rodent models, in neuronal and non-neuronal cell types, we can then verify which of these targets are also regulated by TDP-43 in human ALS/FTD neurons. These mRNA targets act as markers that can reveal how TDP-43 loss of function is associated with neurodegeneration. Further, by identifying common markers, we can exploit them to probe for evidence of TDP-43 dysfunction in various cell models used in research. They may also serve as potential biomarkers of disease and/or targets for therapeutic drugs.

**What has surprised you the most while conducting your research?**

This study is an amalgamation of published TDP-43 knockdown studies and was a pandemic ‘lockdown’ project that started when lab access became limited. As I didn't start my PhD versed in bioinformatics, it was a good opportunity to practice. What surprised me the most was how much I enjoyed it, despite the steep learning curve. When conducting the study, the component of comparing the differential exon usage from published studies was the most interesting to me. As my own research involves TDP-43 knockdown as well, it was always satisfying to see my findings validated in the literature. The findings of this paper have contributed to my research in more ways than I could have imagined, and I hope others in the field will find it useful too, particularly with the supplementary webpage that provides the results in one place.

“The most significant challenge [in ALS research] is uncovering the mechanism of disease to determine effective targets for treatment.”

**Figure:**
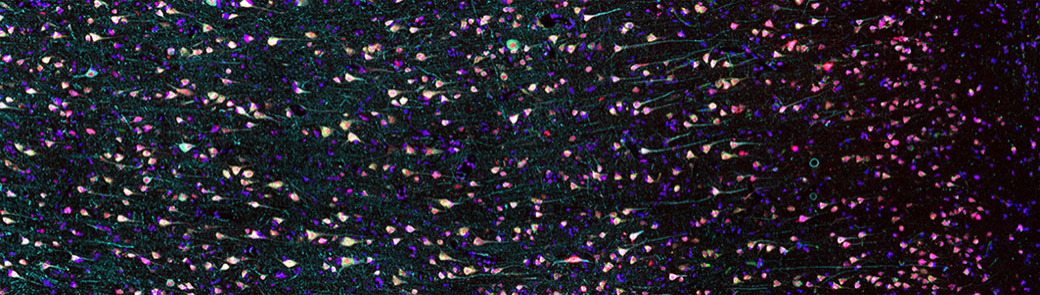
Neurons from post-mortem ALS motor cortex.

**Describe what you think is the most significant challenge impacting your research at this time and how will this be addressed over the next 10 years?**

The heterogeneity in ALS/FTD is one of the biggest hurdles in the field. The increasing number of genetic mutations associated with ALS alone highlights the multitude of cellular processes that could become impaired in disease. Consequently, the most significant challenge is uncovering the mechanism of disease to determine effective targets for treatment. Dysfunctional TDP-43, being the pathological hallmark of disease, certainly holds a lot of promise for uncovering mechanisms. However, as we found, TDP-43 is quite heterogeneous in function too! Over the next few years, ALS will likely move into stratified medicine, where treatment is tailored towards the genetics of the individual.

**What changes do you think could improve the professional lives of early-career scientists?**

From my experience, early-career scientists experience a lot of self-doubt, even when they are motivated and passionate about their research. I think this is brought upon by uncertainty and imposter syndrome when trying to find their ‘niche’ in the space. That is why I think mentorship, career guidance and peer-support is incredibly important.

**What's next for you?**

Currently my most imminent goal is completing my PhD. After that I would like to continue as a postdoctoral researcher. I also have a keen interest in RNA biology and would like to explore that further in my work. Collaborating and working with overseas groups would be another goal of mine, as I enjoy travelling and learning from people with different worldviews.

## References

[DMM049827C1] Cao, M. C. and Scotter, E. L. (2022). Transcriptional targets of amyotrophic lateral sclerosis/frontotemporal dementia protein TDP-43 – meta-analysis and interactive graphical database. *Dis. Model. Mech.* 15, dmm049418. 10.1242/dmm.049418 35946434 PMC9509890

